# Risky Sexual Behaviour among HIV-Infected Adults in Sub-Saharan Africa: A Systematic Review and Meta-Analysis

**DOI:** 10.1155/2023/6698384

**Published:** 2023-04-14

**Authors:** Temesgen Gebeyehu Wondmeneh, Ruhama Gebeyehu Wondmeneh

**Affiliations:** ^1^Department of Public Health, College of Medical and Health Science, Samara University, Semera, Ethiopia; ^2^Departments of Psychology, College of Social Science, Samara University, Semera, Ethiopia

## Abstract

**Background:**

Risky sexual behaviour raises serious public health concerns. The pooled prevalence of risky sexual behaviours among adults living with HIV/AIDS in sub-Saharan Africa was unknown. This systematic review determined the pooled prevalence of risky sexual behaviours and associated factors among HIV-infected adults in sub-Saharan Africa.

**Methods:**

International databases such as PubMed, CINAHL, Google Scholar, and African Journals OnLine were systematically searched to identify articles. The Preferred Reporting Items for Systematic Reviews and Meta-Analysis (PRISMA) guideline was used to conduct the review. All necessary data were extracted independently. Heterogeneity and publication bias were assessed by *I*-squared statistics and Egger's test, respectively. The random-effects meta-analysis model was used to estimate the pooled prevalence. The association between predictors and dependent variable was determined by a pooled odds ratio (OR) with a 95% confidence interval (CI).

**Result:**

In this study, 3713 articles were retrieved from various databases, and 22 of them were included. The pooled prevalence of risky sexual behaviour in sub-Saharan Africa was 36.16% (95% CI: 28.36-44.34) with significant heterogeneity among studies (*I*^2^ = 98.86%, *p* < 0.001). Risky sexual behaviour was significantly associated with the nondisclosure of HIV status (AOR = 1.97, 95% CI: 1.18, 2.76) and alcohol consumption (AOR = 2.29, 95% CI: 1.21, 3.36).

**Conclusion:**

A significant percentage of participants engaged in risky sexual behaviour. Risky sexual behaviour was associated with failure to disclose HIV status and alcohol consumption. Healthcare professionals should advise HIV-positive patients on risk reduction measures like disclosing their HIV status to their sexual partners and avoiding alcohol use in order to promote consistent condom use. *PROSPERO Protocol Registration*. The protocol for this systematic review and meta-analysis has been registered (record ID: CRD42020170967, 09/06/2022).

## 1. Background

There are several definitions of risky sexual behaviour. The most common behavioural definition is unprotected vaginal, oral, or anal intercourse. Risky sexual behaviour can take many forms, ranging from having a large number of sexual partners or engaging in risky sexual activities to engaging in sexual intercourse while under the influence of substances such as alcohol or cocaine [1]. Risky sexual behaviour remains common among people living with HIV [2]. Risky sexual behaviours are a major contributor to the global disease burden [3, 4]. Unsafe sex has been defined as sex between a susceptible person and a partner who has a sexually transmitted infection (STI) without taking infection prevention measures [5]. Peer sexual permissiveness is associated with a greater frequency of risky sexual practices [6]. In most of the world, sexual behaviour plays a major role in the spread of HIV; about 85% of HIV transmission occurs through sexual contact [7]. An estimated 37.9 million people worldwide were infected with HIV in 2018. There were 1.7 million new infections and 770,000 deaths from AIDS [8]. As of 2016, sub-Saharan Africa accounts for about 75% of the global HIV/AIDS prevalence [9]. Although many people engage in safer sex, a significant proportion of seropositive people, ranging from 10% to 60%, continue to engage in unprotected sexual behaviours [10]. In Papua New Guinea, 54% of HIV-positive people did not use condoms [11]. The prevalence of unprotected sex was 37.3% in Thailand [12]. In sub-Saharan Africa, the pooled prevalence of incontinence condom use was 53% in college and university students [13], while the prevalence of current condom nonuse behaviours was 59.8% in adolescents living with HIV/AIDS [14]. In Ethiopia, the pooled prevalence of HIV risk behaviour among people with HIV/AIDS was 34.3% [15]. Condom use has increased almost everywhere, but rates in many developing countries remain low. The wide regional variation suggests that social and economic factors influence sexual behaviour, which has implications for intervention [16, 17]. High-risk sex was linked to lower income, a higher number of sexual partners, and negative attitudes toward condoms [18]. The risk of engaging in sexual practices was higher for those with unstable partners and a low education level [19]. Various studies reported that disclosure of HIV status [20] and discussion of safer sex [21] were protective factors for risky sexual practices. Alcohol users exhibited high-risk sexual behaviour [22–25]. People on ART are not likely to use a condom [26]. Many HIV patients engage in more sexual behaviour as their health improves as a result of the use of ART [27]. The HIV/AIDS epidemic has been determined in relation to its social, economic, and political contexts, as well as other sexually transmitted infections (STIs). However, there are some gaps in understanding the individual's role, such as vulnerability to sexually transmitted disease, which could be a dynamic process involving multiple factors (social, environmental, and cultural) [28]. Despite the high prevalence of HIV/AIDS in sub-Saharan Africa, there is no data on the pooled prevalence of risky sexual behaviour among HIV-infected adults who are a source of HIV infection. As a result, the pooled prevalence of risky sexual behaviour among HIV-infected adults in sub-Saharan Africa was determined in this systematic review, which aids in the development of prevention strategies in the region.

## 2. Methods

### 2.1. Protocol and Registration

A systematic review and meta-analysis were conducted to assess the current magnitude of risky sexual behaviour among HIV-positive adults in sub-Saharan Africa. The Preferred Reporting Items for Systematic Reviews and Meta-Analysis (PRISMA 2020) checklist was used to report this meta-analysis [29] (S1 Table). This protocol has been registered in PROSPERO with ID CRD42020170967.

### 2.2. Searching Strategies

The advanced search strategy was established, built with Boolean operators, and matched to available and accessible databases. All relevant published researches were retrieved from the following major databases: PubMed, CINAHL, Google Scholar, and African Journals OnLine. Additional articles were found by searching the reference lists of the identified studies. We used the following terms and keywords in PubMed and CINAHL to conduct our search: unsafe sex, HIV infections, adults, and Sub-Saharan Africa. The search terms were built up by considering population, exposure, outcome, and context (PEOCo) (details on search strategies can be found in the S1 File).

### 2.3. Selection of Studies for Inclusion in the Review

Two authors (TGW and RGW) implemented the search strategy across relevant databases. Studies identified through different database searches were combined, exported, and managed using the Endnote X8.1 software. Duplicated studies were removed with the Endnote X8.1 software. TGW and RGW independently screened the selected articles for relevance to the review objective using their titles and abstracts. Following the initial screening, the full texts of all studies deemed relevant were retrieved. Two reviewers (TGW and RGW) independently screened the full texts for eligibility, and if there was disagreement, the third expert's colleague reached a consensus.

### 2.4. Operational Definition

#### 2.4.1. Risky Sexual Behaviours

In this review, “risky sexual behaviours” means that those articles reported risky sexual practice as “unprotected sex” to mean sexual activity without the use of a condom or inconsistent condom use with any partner, regardless of HIV serostatus [30].

### 2.5. The Criterion for Selecting Studies for Review

#### 2.5.1. Criteria for Inclusion according to Population, Exposure, Outcome, and Context (PEOCo)

- *Population*. Adults in the age group of 18 years or older were eligible to participate
- *Exposure*. HIV infection
- *Outcome*. Risky sexual behaviours in accordance with the definition [2]
- *Setting*. Studies conducted in sub-Saharan African countries
- *Type of Study*. All descriptive and observational study designs (i.e., cross-sectional, case-control, and cohort) reporting the magnitude of risky sexual behaviours that can be consistent with the PEOCo framework of the Boolean systematic review operators were included
- *Publication Types*. both published and unpublished full text
- Only studies published in English were reviewed
- This meta-analysis comprised studies published between 2012/01/01 and 2022/10/12

#### 2.5.2. Exclusion Criteria

- Studies that failed to provide definitive information about risky sexual behaviours
- Articles with no full text or extraction difficulties were excluded despite contacting the corresponding author (these are excluded because the lack of the full text makes it impossible to evaluate the quality of the article)
- Review, qualitative research, editorials, and commentary were not considered
- Randomized controlled trials (RCTs) and quasiexperimental studies were not used since such types of designs are very limited in this research topic and cannot be consistent with the PEOCo framework of the Boolean systematic review operators
- This review excluded articles with methodological weaknesses as well as review articles

### 2.6. Data Extraction Process

TGW and RGW independently extracted all of the essential data from the primary papers. Disagreements were settled through consensus. The data were retrieved as a summary table in Microsoft Excel using a standardized data extraction format. For each included study, the following information was extracted: the first author's name, the study year, the study country, the sample size, the number of cases with risky sexual behaviour, and the prevalence of risky sexual behaviour. Data for the associated factors were retrieved, and the odds ratio for each factor was calculated using the primary study findings (S2 File).

### 2.7. Quality Assessment

To ensure scientific strength, the Preferred Reporting Items for Systematic Reviews and Meta-Analysis (PRISMA) checklist was employed. The Newcastle-Ottawa Scale for assessing the quality of observational studies is adapted to evaluate the quality of included studies in this systematic review and meta-analysis [31]. The qualities of the included studies were assessed independently by two authors (TGW and RGW). Subjectivities between the two authors were resolved by using the mean score of the reviewers' results, as well as through dialogue and the involvement of other experts. The screened articles' quality and eligibility were evaluated using ten-star ratings in three domain categories. The first is selection, which has 5 stars: sample representativeness (1 star), sampling technique (1 star), response rate (1 star), and exposure ascertainment (2 stars). The second is comparability (2 stars): confounding controlled data/results adjusted for relevant predictors/risk factors (2 stars). The third is outcome (3 stars): assessment of outcome (2 stars) and statistical tests (1 star). Finally, articles with a score of greater or equal to 6 out of 10 were considered high quality and included in the meta-analysis.

### 2.8. Statistical Analysis

The relevant data were extracted using a Microsoft Excel spreadsheet and then exported into the Stata/SE version 15 statistical software for analysis. The included studies were described in the text, graphics, and tables. A separate forest plot was produced to provide summary statistics for studies (e.g., ORs) along with their 95% confidence intervals (95% CIs). Publication bias and heterogeneity were assessed. A graphic test (funnel plot) was used in conjunction with objective tests, such as Egger's statistical test, to determine publication bias [32]. When publication bias exists, nonparametric trim and fill analysis were used to address it [33]. Publication bias was declared when the *p* value is less than 0.05. Cochran's *Q* test was used to assess heterogeneity across studies included in the meta-analysis. Since we are looking at a wide range of study countries, sample sizes, designs, populations, and study periods, we expect the studies to be heterogeneous. As a result, we might assert that we employed a random effects model. Our claim is also consistent with the evidence that heterogeneity in meta-analyses is often unavoidable due to differences in study quality, sample size, technique, and outcome measures. The *I*^2^ test statistic was used to assess the levels of heterogeneity between studies, which indicates the percentage of total variation between studies caused by heterogeneity rather than chance. An *I*^2^ statistic value of 25, 50, and 75% indicates low, moderate, and high heterogeneity, respectively [34]. We did a sensitivity analysis to determine the extent to which changes in the analysis techniques affect the meta-analytical results and conclusions [35]. This aids in determining the robustness of the study's conclusion as well as the effect of methodological quality, sample size, and analysis methodologies on meta-analytical results. The leave-one-out sensitivity analysis was performed in particular to analyze the expected influence of studies on the pooled estimates in meta-analyses. The new pool of risky sexual practice was then compared to the original pool of risky sexual practice. If the new pooled risky sexual practice failed outside the 95% confidence interval (CI) of the previous pooled risky sexual practice, we concluded that the excluded study has a substantial effect on the study and should be excluded from the final analysis. Subgroup analysis was undertaken by division of African region, country, and ART use. Metan command was used to compute the pooled odds ratio (OR) by using the odds ratio (OR) with 95% confidence intervals (CI) of each factor to determine the association between dependent and independent factors. The selected articles and findings were then summarized using tables and forest plots.

### 2.9. Ethical Approval

A systematic review and meta-analysis use published data and do not require ethical approval.

## 3. Results

### 3.1. Study Selection and Identification

**Figure 1:**
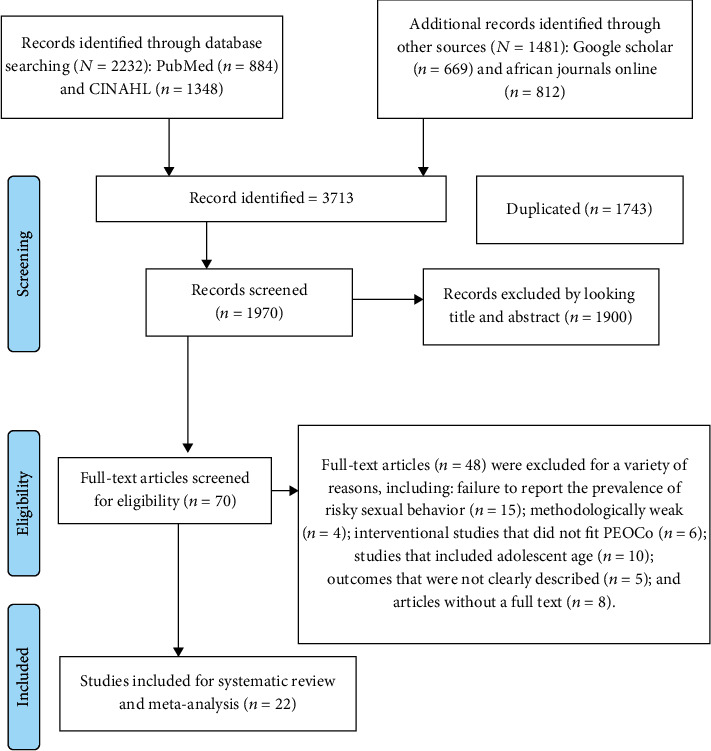
PRISMA flow diagram which shows the selection of articles for systematic review and meta-analysis.

Searching electronic databases (PubMed, CINAHL, Google Scholar, and African Journals OnLine) yielded 3713 articles. 1743 articles were removed due to duplication. After reading their titles and abstracts, 1900 articles were excluded since they were irrelevant to this review. Forty-eight papers were removed for a variety of reasons, including failure to report the prevalence of risky sexual behaviours, methodologically weak studies, interventional studies that did not fit PEOCo, studies that included adolescent age, outcomes that were not clearly described, and articles without a full text. Finally, 22 articles were included in this systematic review and meta-analysis, as illustrated in the PRISMA flow diagram (Figure 1).

### 3.2. Characteristics of the Included Studies

After an in-depth assessment of the papers, 22 relevant primary studies were ascertained to be eligible for inclusion in this systematic review and meta-analysis. All of the studies included were published between 2012/01/01 and 2022/10/12. Ten sub-Saharan countries were represented in this review. Of these 22 relevant articles, ten were conducted in Ethiopia; two were from Kenya, Nigeria, and Uganda; and one was from Togo, Ghana, Botswana, Namibia, Tanzania, and South Africa. All of the included articles used a cross-sectional study design. A total of 12415 participants who were 18 years of age or older were included in the current meta-analysis. The smallest and largest sample sizes were 126 and 1196, respectively [36, 37]. A minimum of 12 percent of risky sexual behaviours was reported in the studies conducted in Botswana [38] and Namibia [37], while a study conducted in Uganda reported a maximum of 81% [39]. Almost all studies had a high response rate (≥91%), which could be attributed in part to data collection using interviewer-administered questionnaires (Table 1).

### 3.3. Quality of the Included Study

During the quality assessment, all of the included studies had reliable methodological quality (the Newcastle-Ottawa Scale for cross-sectional study quality assessment). The quality scores of the included articles ranged from 7 to 10, with an average quality score of 8.875 (SD = 1.191). Studies with a quality score of ≥ 6 were deemed to be of high quality. Finally, all 22 articles included have a quality score of 7 and higher (S3 File).

### 3.4. The Pooled Prevalence of Risky Sexual Behaviours among HIV-Infected Adults

**Figure 2:**
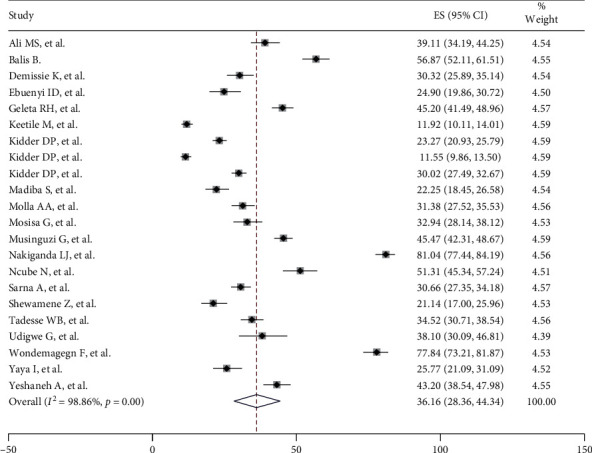
Forest plot of the pooled prevalence of HIV risk behaviour among HIV-infected adults in sub-Saharan Africa.

The overall pooled prevalence of risky sexual behaviours among adults living with HIV/AIDS in sub-Saharan Africa was 36.16% (95% CI: 28.36; 44.34). There was significant heterogeneity among studies (*I*^2^ = 98.86%, *p* < 0.001). As a result, the random effects model was used to estimate the DerSimonian and Laird overall effect (Figure 2).

### 3.5. Subgroup Analysis of Risky Sexual Behaviours among HIV-Infected Adults

Subgroup analysis was carried out based on the African region subdivision, country level, and ART use. The subgroup analysis by subdivision of African region revealed that East Africa had the highest risky sexual behaviour (41.42%; 95% CI: 32.79%, 50.36%) and the highest level of heterogeneity (*I*^2^ = 98.61%, *p* < 0.001), followed by West Africa, in which the prevalence of risky sexual behaviour is 34.63% (95% CI: 22.27%, 48.15%), with a significant high level of heterogeneity (*I*^2^ = 94.31%, *p* < 0.001). According to the country-level subgroup analysis, Uganda had the highest prevalence of risky sexual behaviours, at 59% (95% CI: 56%, 61%), while Botswana and Namibia had the lowest (12%). However, due to low heterogeneity (*I*^2^ = 0.00), we are unable to consider these single or two studies. Only the studies conducted in Ethiopia showed a significant level of heterogeneity (*I*^2^ = 97.7%, *p* < 0.001). A subgroup analysis by ART use was also done, and it was found that ART users had higher rates of risky sexual behaviour (38.11%; 95% CI: 27.69%, 49.11%), and the reported heterogeneity was a significant high (*I*^2^ = 98.86%, *p* < 0.001). For ART nonusers, the single study prevalence of risky sexual behaviour was 30.66% (95% CI: 27.35%, 34.18%) with no heterogeneity (*I*^2^ = 0.0%) (Table 2).

### 3.6. Publication Bias

**Figure 3:**
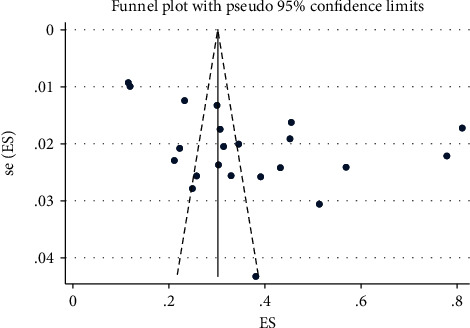
A funnel plot of publication bias for the pooled prevalence of risky sexual behaviour in HIV-infected adults in sub-Saharan Africa.

Egger's test was used to check for publication bias among the 22 included studies in the meta-analysis. No evidence of publication bias was found using Egger's test (*β* = 0.046, SE = 0.1, *p* = 0.65). The funnel plots' shape demonstrates the asymmetric distribution of the effect estimates (Figure 3).

### 3.7. Sensitivity Analysis

**Figure 4:**
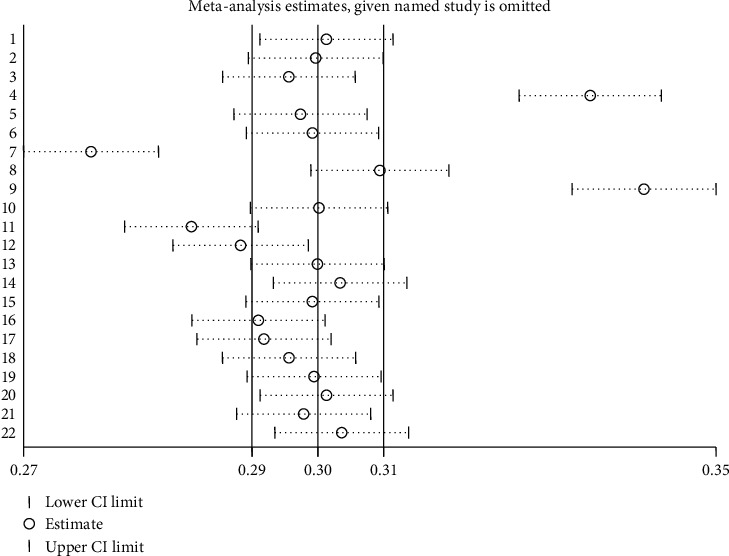
The sensitivity analysis of the prevalence of each study being removed one at a time in sub-Saharan Africa.

A sensitivity analysis was carried out to see if any small study effects influenced the pooled effect size. The leave-one-out sensitivity analysis revealed no significant differences (Figure 4). The findings of the current meta-analysis can be viewed as stable.

### 3.8. Determinants of Risky Sexual Behaviours among HIV-Infected Adults

In this systematic review and meta-analysis, nondisclosure of HIV status and alcohol consumption were found to be statistically significant risk factors for risky sexual behaviours.

### 3.9. The Association between HIV Nondisclosure and Risky Sexual Behaviours

**Figure 5:**
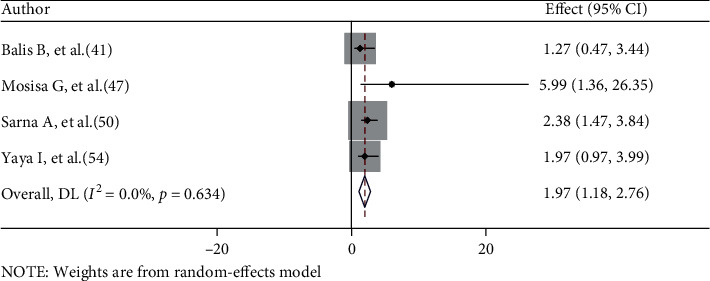
The association between HIV nondisclosure and risky sexual behaviours.

Four studies were included to determine the relationship between HIV status nondisclosure and risky sexual behaviours [41, 47, 50, 54]. Two studies [41, 54] found no significant association between nondisclosure and risky sexual behaviours, whereas the other two studies [47, 50] found that nondisclosure is a risk factor for risky sexual behaviours. The random effects meta-analysis found that patients who did not disclose their HIV status to their sexual partner were 1.97 times more likely to engage in risky sexual behaviours than those who did (AOR = 1.97, 95% CI: 1.18–2.76). There was no heterogeneity among the included studies (*I*^2^ = 0.0%, *p* = 0.634) (Figure 5).

### 3.10. The Association between Alcohol Consumption and Risky Sexual Behaviour

**Figure 6:**
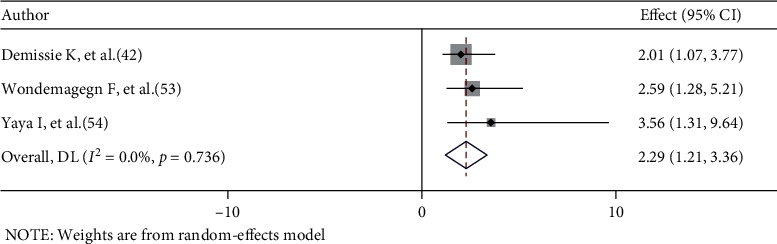
The association between alcohol consumption and risky sexual behaviour.

Three studies were identified to determine the association between alcohol consumption and risky sexual behaviour [42, 53, 54]. All of these studies' findings revealed a significant association between alcohol consumption and risky sexual behaviour. In this meta-analysis, HIV-infected adults who consumed alcohol were 2.29 times more likely to engage in risky sexual behaviours than those who could not consume alcohol (AOR = 2.29, 95% CI: 1.21–3.36). There was no heterogeneity between the included studies (*I*^2^ = 0.0%, *p* = 0.736) (Figure 6).

## 4. Discussion

In order to prevent the recent rise in risky sexual behaviour, which is a major global disease burden [3, 4], that contributes to the expansion of STIs such as HIV/AIDS [7–10], particularly in sub-Saharan Africa, adequately recognizing the individual role that is being risky, which depends on social, environmental, and cultural factors [28], could be important. In this meta-analysis, the pooled prevalence of risky sexual behaviours among adults living with HIV/AIDS in sub-Saharan Africa was 36.16% (95% CI: 28.36, 44.34). This result is somewhat in line with an earlier study in Thailand [12]. However, the current finding is significantly lower than an earlier international study [10] and a Papua New Guinea study [11]. Differences in study settings, differences in the definition used to define risky sexual behaviour [1], variations in socioeconomic status and educational status, and cultural and contextual factors could all explain the variation [16–19, 28]. Another variation may be the differing concerns of governmental and nongovernmental organizations in HIV prevention and control across countries.

Compared to previous studies on sub-Saharan Africa [13, 14], the current finding is significantly lower. Different study populations, such as adolescents [14] and college and university students [13], may also contribute to the variation because these populations are frequently exposed to risky sexual behaviours as a result of peer sexual pressure [6] and their age level. Since risk sexual behaviour is a sensitive issue, the data collection method may also contribute for these variations. However, the present finding is slightly in line with a study conducted in Ethiopia [15]. In the subgroup analysis by African subdivision, East Africa had the highest risky sexual behaviour (41.42%), followed by West Africa (34.63%), while at the country-level subgroup analysis, Uganda had the highest prevalence of risky sexual behaviours (59%). This variation could be attributed to various HIV-risk sexual prevention strategies that have been implemented in countries, the expansion of commercial sex workers, and HIV prevention practice negligence. Therefore, experts responsible for disease prevention should focus on HIV counselling, support, and awareness creation through various media such as radio, television, posters, leaflets, banners, conferences, and research. Another key variation may be due to differences in measuring risky sexual behaviour [1] and substantial regional differences in the social and economic factors affecting sexual behaviour [16, 17]. The variation in the country-level subgroup analysis could also be due to the presence of only one or two studies in these countries with insufficient sample sizes to determine the outcome. Antiretroviral therapy (ART) users had high rates of risky sexual behaviour (38.11%). This evidence is consistent with a previous finding [26]. This may be because as the health of people living with HIV improves as a result of continuing ART use, many resume sexual activities [27]. HIV-positive people who did not disclose their HIV serostatus to their sexual partners were more likely to engage in risky sexual behaviour than HIV-positive people who did disclose their HIV serostatus. This finding is consistent with a prior study [20]. This may be due to the fact that HIV-positive adults who were unaware of the serostatus of their sexual partners were less likely to use a condom during a sexual relationship. Disclosing HIV status is a vital part of HIV prevention because it encourages partners to be aware of each other's HIV status, encourages them to get tested for HIV, and increases adherence to therapy and follow-up care. More health education interventions, such as extended adherence counselling and education about the benefits of disclosure for all HIV-infected adults, are needed to increase the rate of HIV status disclosure and reduce high-risk sexual behaviour among adults living with HIV/AIDS. An essential component of preventing HIV spread is safer sex [21], which can be achieved through disclosure. HIV persons' interpersonal communications, particularly about disclosing their HIV status to sexual partners, are a critical component of HIV transmission prevention. Counselling during HIV testing and follow-up care about disclosing their HIV status should be strengthened to reduce sexually risky behaviours. HIV-infected adults who consumed alcohol were more likely to engage in HIV-risk behaviours than their nonalcoholic counterparts. This is in line with the findings of previous studies [22–25]. This may be due to the fact that alcohol can reduce an individual's perception of the risk of HIV transmission and impair thinking and decision-making abilities about safe sex, due to alcohol's constrained effect on cognitive capacity, causing someone to focus only on motivating their immediate senses. Thus, alcohol intake puts HIV patients at risk for having unstable partners [18, 19] and for engaging in sexual activity without taking any HIV prevention measures [5]. Given the strong relationship between alcohol use and HIV risk behaviour, clinicians must incorporate alcohol counselling into HIV care for HIV-infected adults. Policies and strategies aimed at reducing alcohol consumption and HIV risk behaviour among adults living with HIV/AIDS must also be implemented.

It is limited to English-language articles and may not represent research published in other languages. Due to the cross-sectional nature of all included studies, it is impossible to establish a cause-and-effect relationship between risky sexual behaviour and risk factors. Another limitation for this study is that a large value of *I*^2^ indicates that heterogeneity exists among the studies. Even after performing subgroup analyses, there was high heterogeneity across all analyses, indicating that these study variables could not explain everything. The existence of heterogeneity could be due to differences in participants among studies. Moreover, the study was conducted in only a few sub-Saharan African countries, which may not be representative of the rest of the countries in sub-Saharan Africa. Despite some limitations, this was the first systematic review and meta-analysis to assess the pooled prevalence of risky sexual behaviour among adults living with HIV/AIDS in sub-Saharan Africa. Understanding the magnitude of risky sexual behaviour and its determinants, which is a major cause of HIV expansion [7–9], can aid in policy implementation in the region, which is critical to reducing the current HIV epidemic in sub-Saharan Africa.

## 5. Conclusions

A significant percentage of adults living with HIV/AIDS practised risky sexual behaviour in sub-Saharan Africa. East Africa had the highest risky sexual behaviour. ART users also had a high rate of risky sexual behaviour. HIV status nondisclosure and alcohol consumption were significant risk factors for risky sexual behaviour. To reduce high-risk sexual behaviour, HIV care services such as strict adherence and counselling should improve consistent condom use. A common HIV prevention practice should exist across all nations and be strengthened. HIV-infected adults with alcohol use disorders and those who have not disclosed their HIV status to their partners should be screened and treated as part of the routine HIV treatment, care, and support package.

## Figures and Tables

**Table 1:** The characteristics of the included studies in the meta-analysis, based on country, publication year, sample size, prevalence, and response rate of risky sexual behaviour in sub-Saharan Africa (*n* = 22).

ID	Authors	Country	Publication year	Sample size	Risky sexual behaviours		Response rate
					*n*	%	
1.	Ali et al. [40]	Ethiopia	2019	358	140	39.1	91%
2.	Balis [41]	Ethiopia	2020	422	240	56.9	97.7%
3.	Demissie et al. [42]	Ethiopia	2015	376	114	30.3	100%
4.	Ebuenyi et al. [43]	Nigeria	2017	241	60	24.9	100%
5.	Geleta and Tiruneh [44]	Ethiopia	2020	677	306	45.2	100%
6.	Keetile and Kgosidintsi [38]	Botswana	2018	1065	127	11.9	100%
7.	Kidder et al. [37]	Kenya	2013	1156	269	23.3	93.6%
8.	Kidder et al. [37]	Namibia	2013	1186	137	11.6	93.6%
9.	Kidder et al. [37]	Tanzania	2013	1196	359	30	93.6%
10.	Madiba and Letsoalo [45]	South Africa	2014	400	89	22.3	100%
11.	Molla and Gelagay [46]	Ethiopia	2017	513	161	31.4	99%
12.	Mosisa et al. [47]	Ethiopia	2018	337	111	32.9	100%
13.	Musinguzi et al. [48]	Uganda	2014	939	427	45.5	97.8%
14.	Nakiganda et al. [39]	Uganda	2017	517	419	81	100%
15.	Ncube et al. [49]	Ghana	2012	267	137	51.3	100%
16.	Sarna et al. [50]	Kenya	2012	698	214	30.7	96.9%
17.	Shewamene et al. [51]	Ethiopia	2015	317	67	21.1	100%
18.	Tadesse and Gelagay [52]	Ethiopia	2019	562	194	34.5	100%
19.	Udigwe et al. [36]	Nigeria	2014	126	48	38.1	100%
20.	Wondemagegn and Berkessa [53]	Ethiopia	2020	352	274	77.8	97.8%
21.	Yaya et al. [54]	Togo	2014	291	75	25.8	91.8%
22.	Yeshaneh et al. [55]	Ethiopia	2021	419	181	43.2	100%

**Table 2:** A subgroup analysis of the prevalence of risky sexual behaviour in sub-Saharan Africa based on a random effects model (*n* = 22).

Variables	Categories	Included study	Sample size	Estimated	Heterogeneity
				Prevalence (95% CI)	*I* ^2^ (%), *p* value
Region	East Africa	15	8839	41.42 (32.79, 50.36)	98.61, *p* < 0.001
	West Africa	4	925	34.63 (22.27, 48.15)	94.31, *p* < 0.001
	South Africa	3	2651	14.73 (9.89, 20.34)	—

Countries	Togo	1	291	26 (21, 31)	—
	Kenya	2	1854	26 (24, 28)	—
	Ghana	1	267	51 (45,57)	—
	Botswana	1	1065	12 (10,14)	—
	Ethiopia	10	4333	41 (32, 51)	97.6, *p* < 0.001
	Nigeria	2	367	29 (25, 34)	—
	Uganda	2	1456	59 (56, 61)	—
	Namibia	1	1186	12 (10, 13)	—
	Tanzania	1	1196	30 (27, 33)	—
	South Africa	1	400	22 (18, 27)	—

ART use	Yes	16	6973	38.11 (27.69, 49.11)	98.86, *p* < 0.001
	No	1	698	30.66 (27.35, 34.18)	—

Em dash (—) indicates no heterogeneity.

## Data Availability

All data generated or analyzed during this study are included in this article.

## Abbreviations

HIV: Human immunodeficiency virus
AIDS: Acquired immunodeficiency syndrome
ART: Antiretroviral treatment
AOR: Adjusted odds ratio
CI: Confidence interval
S: Supplementary
PEOCo: Population, exposure, outcome, and context.

## Supplementary Materials

Supplementary 1 S1 Table: checklist of Preferred Reporting Items for Systematic Reviews and Meta-Analysis (PRISMA 2020) for risky sexual behaviour among HIV-infected adults in sub-Saharan Africa: a systematic review and meta-analysis.

Supplementary 2 S1 File: the detailed search strategy for the risky sexual behaviour among HIV-infected adults in sub-Saharan Africa.

Supplementary 3 S2 File: data extraction sheet sorted by country, sample size, publication year, and prevalence of risky sexual behaviour for HIV-infected adults in sub-Saharan Africa.

Supplementary 4 S3 File: quality assessment of the included cross-sectional studies using the modified Newcastle-Ottawa Scale for systematic review and meta-analysis of risky sexual behaviour among HIV-infected adults in sub-Saharan Africa.
