# Supplementary material for: Risky Sexual Behaviour among HIV-Infected Adults in Sub-Saharan Africa: A Systematic Review and Meta-Analysis
Source: Biomed Res Int. 2023 Apr 14;2023:6698384. doi: 10.1155/2023/6698384 (PMC10643038; doi:10.1155/2023/6698384)
Supplement: Supplementary 2 — S1 File: the detailed search strategy for the risky sexual behaviour among HIV-infected adults in sub-Saharan Africa. [file 6698384.f2.docx]

S1 File. The detailed search strategy for the risky sexual behavior among HIV-infected adults in Sub-Saharan Africa.

| **PubMed** | | |
| --- | --- | --- |
| Population | | (Adult [All Fields] OR Adult [Mesh Terms] OR Adults [All Fields] OR Young Adult [Mesh Terms] OR Young Adult [All Fields] OR (Adult and Young) [All Fields] OR (Adults and Young) [All Fields] OR Young Adults) |
| **AND** | | |
| Exposure | | (HIV Infections [Mesh Terms] OR HIV Infections [All Fields] OR HIV Infection [All Fields] OR (Infection and HIV) [All Fields] OR (Infections and HIV) [All Fields] OR HTLV-III-LAV Infections [All Fields] OR HTLV III LAV Infections [All Fields] OR HTLV-III-LAV Infection [All Fields] OR (Infection and HTLV-III-LAV) [All Fields] OR (Infections and HTLV-III-LAV) [All Fields] OR T-Lymphotropic Virus Type III Infections and Human [All Fields] OR (T Lymphotropic Virus Type III Infections and Human) [All Fields] OR HTLV-III Infections [All Fields] OR HTLV III Infections [All Fields] OR HTLV-III Infection [All Fields] OR (Infection and HTLV-III) [All Fields] OR (Infections and HTLV-III) [All Fields] OR HIV Coinfection [All Fields] OR (Coinfection and HIV) [All Fields] OR (Coinfections and HIV) [All Fields] OR HIV Coinfections) [All Fields]) |
| **AND** | | |
| Outcome | (Unsafe Sex [Mesh Terms] OR Unsafe Sex [All Fields] OR (Sex and Unsafe) [All Fields] OR Unprotected Sex [All Fields] OR (Sex and Unprotected) [All Fields] OR High-Risk Sex [All Fields] OR High Risk Sex [All Fields] OR (Sex and High-Risk) OR Unprotected Intercourse OR (Intercourse and Unprotected) [All Fields] OR Condom less Sex [All Fields] OR (Sex and Condom less) [All Fields] ) | |
| **AND** | | |
| Context | (Africa South of the Sahara [Mesh Terms] OR Africa South of the Sahara [All Fields] OR Sub-Saharan Africa [All Fields] OR Subsaharan Africa [All Fields] OR (Africa and Sub-Saharan) [All Fields]) | |
| Filters | Humans, English, Free full text, publication date from 2012/01/01 - 2022/10/12 | |
| Date and time | 2022-09-10, 3:55 PM | |
| Total articles | 884 | |
| **CINAHL** | | |
| Population | Adults [All Fields] | |
| **AND** | | |
| Exposure | HIV Infection [All Fields] | |
| **AND** | | |
| Outcome | Unsafe Sex[All Fields] | |
| **AND** | | |
| Context | Sub-Saharan Africa [All Fields] | |
| Filter | English, Publication date from 2012/01/01 – 2022/10/12 | |
| Date and time | 2022-09- 16, 4:30PM | |
| Total articles | 1348 | |
| **Google scholar** | | |
| Population | Adults | |
| **AND** | | |
| Exposure | HIV/AIDS ("HIV infection") | |
| **AND** | | |
| Outcome | "Unsafe sex"("risky sexual practice”) | |
| **AND** | | |
| Context | “Sub-Saharan Africa” | |
| filter | Publication date: 2012 – 2022 | |
| Date and time | 2022-10-03, 5:01PM | |
| Total articles | 669 | |
| **African Journals Online: using the following keywords in the title** | | |
| Population | Adults | |
| Exposure | HIV infection OR HIV/AIDS | |
| Outcome | Unsafe sex OR risky sexual practice | |
| Date and time | 2022-10-11, 5: 30 PM | |
| Total articles | 812 | |
