# Supplementary material for: Risky Sexual Behaviour among HIV-Infected Adults in Sub-Saharan Africa: A Systematic Review and Meta-Analysis
Source: Biomed Res Int. 2023 Apr 14;2023:6698384. doi: 10.1155/2023/6698384 (PMC10643038; doi:10.1155/2023/6698384)
Supplement: Supplementary 3 — S2 File: data extraction sheet sorted by country, sample size, publication year, and prevalence of risky sexual behaviour for HIV-infected adults in sub-Saharan Africa. [file 6698384.f3.docx]

S2 File. Data extraction sheet sorted by country, sample size, publication year, and prevalence of risky sexual behavior for HIV-infected adults in Sub-Saharan Africa.

| **Study ID** | **Author** | **Publication year** | **Country** | **Sample size** | **Cases** | **Prevalence** |
| --- | --- | --- | --- | --- | --- | --- |
|  | Ali MS,et al.(40) | 2019 | Ethiopia | 358 | 140 | 39.10% |
|  | Balis B.(41) | 2020 | Ethiopia | 422 | 240 | 56.90% |
|  | Demissie K, et al.(42) | 2015 | Ethiopia | 376 | 114 | 30.30% |
|  | Ebuenyi ID, et al.(43) | 2017 | Nigeria | 241 | 60 | 24.90% |
|  | Geleta RH,et al.(44) | 2020 | Ethiopia | 677 | 306 | 45.20% |
|  | Keetile M,et al.(38) | 2018 | Botswana | 1065 | 127 | 11.90% |
|  | Kidder DP,et al.(37) | 2013 | Kenya | 1156 | 269 | 23.30% |
|  | Kidder DP,et al.(37) | 2013 | Namibia | 1186 | 137 | 11.55% |
|  | Kidder DP,et al.(37) | 2013 | Tanzania | 1196 | 359 | 30.00% |
|  | Madiba S, et al.(45) | 2014 | South Africa | 400 | 89 | 22.25% |
|  | Molla AA, et al.(46) | 2017 | Ethiopia | 513 | 161 | 31% |
|  | Mosisa G,et al.(47) | 2018 | Ethiopia | 337 | 111 | 32.90% |
|  | Musinguzi G,et al.(48) | 2014 | Uganda | 939 | 427 | 45.50% |
|  | Nakiganda LJ,et al.(39) | 2017 | Uganda | 517 | 419 | 81% |
|  | Ncube N,et al.(49) | 2012 | Ghana | 267 | 137 | 51.30% |
|  | Sarna A, et al. (50) | 2012 | Kenya | 698 | 214 | 30.66% |
|  | Shewamene Z, et al.(51) | 2015 | Ethiopia | 317 | 67 | 21.10% |
|  | Tadesse WB, et al.(52) | 2019 | Ethiopia | 562 | 194 | 34.50% |
|  | Udigwe G,et al.(36) | 2014 | Nigeria | 126 | 48 | 38.10% |
|  | Wondemagegn F, et al.(53) | 2020 | Ethiopia | 352 | 274 | 77.80% |
|  | Yaya I, et al.(54) | 2014 | Togo | 291 | 75 | 25.80% |
|  | Yeshaneh A,et al.(55) | 2021 | Ethiopia | 419 | 181 | 43.20% |
