# Supplementary material for: Risky Sexual Behaviour among HIV-Infected Adults in Sub-Saharan Africa: A Systematic Review and Meta-Analysis
Source: Biomed Res Int. 2023 Apr 14;2023:6698384. doi: 10.1155/2023/6698384 (PMC10643038; doi:10.1155/2023/6698384)
Supplement: Supplementary 4 — S3 File: quality assessment of the included cross-sectional studies using the modified Newcastle-Ottawa Scale for systematic review and meta-analysis of risky sexual behaviour among HIV-infected adults in sub-Saharan Africa. [file 6698384.f4.docx]

S3 File. Quality assessment of the included cross-sectional studies using the modified Newcastle–Ottawa scale for systematic review and meta-analysis of risky sexual behaviour among HIV-infected adults in Sub-Saharan Africa

**Title: Risky sexual behavior among HIV-infected adults in Sub-Saharan Africa: A systematic review and meta-analysis**

The quality of each study was assessed by two authors (TG and RG) independently, and an average score of 6 or above was used as a cut point for the inclusion of research articles. The differences in the results were settled by taking the mean score of the results of both reviewers, through discussion and dialogue with a third expert. The three domains of categories are listed below, along with their subsections and rating stars.

## Selection:

1. Representativeness of the sample:
   1. Truly representative of the average in the target population. * (all subjects or random sampling)
   2. Somewhat representative of the average in the target group. * (non-random sampling)
   3. Selected group of users/convenience sample.
   4. No description of the derivation of the included subjects.
2. Sample size:
   1. Justified and satisfactory (including sample size calculation). *
   2. Not justified.
   3. No information provided
3. Non-respondents:
   1. Proportion of target sample recruited attains pre-specified target or basic summary of non-respondent characteristics in sampling frame recorded. *
   2. Unsatisfactory recruitment rate, no summary data on non-respondents.
   3. No information provided
4. Ascertainment of the exposure (risk factor):
   1. Vaccine records/vaccine registry/clinic registers/hospital records only. **
   2. Parental or personal recall and vaccine/hospital records.*
   3. Parental/personal recall only.

**Comparability:** (Maximum 2 stars)

1. Comparability of subjects in different outcome groups on the basis of design or analysis. Confounding factors controlled.

## Outcome:

1. Data/ results adjusted for relevant predictors/risk factors/confounders e.g. age, sex, time since vaccination, etc. **
2. Data/results not adjusted for all relevant confounders/risk factors/information not provided.
   1. Assessment of outcome:
      1. Independent blind assessment using objective validated laboratory methods.**
      2. Unblindedassessment using objective validated laboratory methods. **
      3. Used non-standard or non-validated laboratory methods with gold standard. *
      4. No description/non-standard laboratory methods used.
   2. Statistical test:
      1. Statistical test used to analyse the data clearly described, appropriate and measures of association presented including confidence intervals and probability level (p value). *
      2. Statistical test not appropriate, not described or incomplete.

# This scale has been adapted from the Newcastle-Ottawa Quality Assessment Scale for cohort studies to provide quality assessment of cross sectional studies^1^.

Reference: ^1^Herzog R, et al. Is Healthcare Workers’ Intention to Vaccinate Related to their Knowledge, Beliefs and Attitudes? A Systematic Review.*BMC Public Health*2013**13**:154

# S3 File displays the quality of included studies as determined by the Newcastle-Ottawa Scale Appraisal Checklist.

| Authors | Publication year | Reviewers | Selection | | | | Comparability | Outcome | | Total |
| --- | --- | --- | --- | --- | --- | --- | --- | --- | --- | --- |
|  |  |  | 1 | 2 | 3 | 4 |  | 1 | 2 |  |
| Yaya I, et al. (40) | 2014 | Temesgen  Gebeyehu (TG) |  |  | ⁎ | ⁎⁎ | ⁎⁎ | ⁎⁎ | ⁎ | 8 |
|  |  | Ruhama  Gebeyehu (RG) |  |  | ⁎ | ⁎⁎ | ⁎⁎ | ⁎⁎ | ⁎ | 8 |
|  |  | Average score |  |  | 1 | 2 | 2 | 2 | 1 | 8 |
| Sarna A, et al. (41) | 2012 | Temesgen  Gebeyehu (TG) |  |  | ⁎ | ⁎⁎ | ⁎⁎ | ⁎⁎ | ⁎ | 8 |
|  |  | Ruhama  Gebeyehu (RG) |  |  | ⁎ | ⁎⁎ | ⁎⁎ | ⁎⁎ | ⁎ | 8 |
|  |  | Average score |  |  | 1 | 2 | 2 | 2 | 1 | 8 |
| Ncube N, et al. (42) | 2012 | Temesgen  Gebeyehu (TG) |  |  | ⁎ | ⁎⁎ | ⁎⁎ | ⁎⁎ | ⁎ | 8 |
|  |  | Ruhama  Gebeyehu (RG) |  |  | ⁎ | ⁎⁎ | ⁎⁎ | ⁎⁎ | ⁎ | 8 |
|  |  | Average score |  |  | 1 | 2 | 2 | 2 | 1 | 8 |
| Keetile M, et al. (38) | 2018 | Temesgen  Gebeyehu (TG) | ⁎ | ⁎ | ⁎ | ⁎⁎ |  | ⁎⁎ |  | 7 |
|  |  | Ruhama  Gebeyehu (RG) | ⁎ | ⁎ | ⁎ | ⁎⁎ |  | ⁎⁎ |  | 7 |
|  |  | Average score | 1 | 1 | 1 | 2 |  | 2 |  | 7 |
| Ali MS,et al. (43) | 2019 | Temesgen  Gebeyehu (TG) | ⁎ | ⁎ | ⁎ | ⁎⁎ | ⁎⁎ |  | ⁎ | 8 |
|  |  | Ruhama  Gebeyehu (RG) | ⁎ | ⁎ | ⁎ | ⁎⁎ | ⁎⁎ | ⁎ | ⁎ | 9 |
|  |  | Average score | 1 | 1 | 1 | 2 | 2 | 0.5 | 1 | 8.5 |
| Udigwe G, et al. (36) | 2014 | Temesgen  Gebeyehu (TG) |  |  | ⁎ | ⁎⁎ | ⁎⁎ | ⁎⁎ |  | 7 |
|  |  | Ruhama  Gebeyehu (RG) |  |  | ⁎ | ⁎⁎ | ⁎⁎ | ⁎⁎ | ⁎ | 8 |

|  |  | Average score |  |  | 1 | 2 | 2 | 2 | 0.5 | 7.5 |
| --- | --- | --- | --- | --- | --- | --- | --- | --- | --- | --- |
| Nakiganda LJ, et al. (39) | 2017 | Temesgen  Gebeyehu (TG) | ⁎ |  | ⁎ | ⁎⁎ | ⁎⁎ | ⁎⁎ | ⁎ | 9 |
|  |  | Ruhama  Gebeyehu (RG) | ⁎ |  | ⁎ | ⁎⁎ | ⁎⁎ | ⁎⁎ | ⁎ | 9 |
|  |  | Average score | 1 |  | 1 | 2 | 2 | 2 | 1 | 9 |
| Kidder DP, et al. (37) | 2013 | Temesgen  Gebeyehu (TG) | ⁎ | ⁎ |  | ⁎⁎ |  | ⁎⁎ | ⁎ | 7 |
|  |  | Ruhama  Gebeyehu (RG) | ⁎ | ⁎ | ⁎ | ⁎⁎ |  | ⁎⁎ | ⁎ | 8 |
|  |  | Average score | 1 | 1 | 0.5 | 2 |  | 2 | 1 | 7.5 |
| Wondemagegn F, et al. (44) | 2020 | Temesgen  Gebeyehu (TG) | * | * | * | ** | ** | ** | * | 10 |
|  |  | Ruhama  Gebeyehu (RG) | * | * | * | ** | ** | ** | * | 10 |
|  |  | Average score | 1 | 1 | 1 | 2 | 2 | 2 | 1 | 10 |
| Musinguzi G, et al. (45) | 2014 | Temesgen  Gebeyehu (TG) | * | * | * | ** | ** | ** | * | 10 |
|  |  | Ruhama  Gebeyehu (RG) | * | * | * | ** | ** | ** | * | 10 |
|  |  | Average score | 1 | 1 | 1 | 2 | 2 | 2 | 1 | 10 |
| Demissie K, et al. (46) | 2015 | Temesgen  Gebeyehu (TG) | * | * | * | ** | ** | ** | * | 10 |
|  |  | Ruhama  Gebeyehu (RG) | * | * | * | ** | ** | ** | * | 10 |
|  |  | Average score | 1 | 1 | 1 | 2 | 2 | 2 | 1 | 10 |
| Shewamene Z, et al. (47) | 2015 | Temesgen  Gebeyehu (TG) | * | * | * | ** | ** | ** | * | 10 |
|  |  | Ruhama  Gebeyehu (RG) | * | * | * | ** | ** | ** | * | 10 |
|  |  | Average score | 1 | 1 | 1 | 2 | 2 | 2 | 1 | 10 |
| Mosisa G, et al. (48) | 2018 | Temesgen  Gebeyehu (TG) | * | * | * | ** | ** | ** | * | 10 |

|  |  | Ruhama  Gebeyehu (RG) | * | * | * | ** | ** | ** | * | 10 |
| --- | --- | --- | --- | --- | --- | --- | --- | --- | --- | --- |
|  |  | Average score | 1 | 1 | 1 | 2 | 2 | 2 | 1 | 10 |
| Balis B, et al. (49) | 2020 | Temesgen  Gebeyehu (TG) | * | * | * | ** | ** | ** | * | 10 |
|  |  | Ruhama  Gebeyehu (RG) | * | * | * | ** | ** | ** | * | 10 |
|  |  | Average score | 1 | 1 | 1 | 2 | 2 | 2 | 1 | 10 |
| Geleta RH, et al. (50) | 2020 | Temesgen  Gebeyehu (TG) | * | * | * | ** | ** | ** | * | 10 |
|  |  | Ruhama  Gebeyehu (RG) | * | * | * | ** | ** | ** | * | 10 |
|  |  | Average score | 1 | 1 | 1 | 2 | 2 | 2 | 1 | 10 |
| Yeshaneh A, et al. (51) | 2021 | Temesgen  Gebeyehu (TG) | * | * | * | ** | ** | * | * | 9 |
|  |  | Ruhama  Gebeyehu (RG) | * | * | * | ** | ** | ** | * | 10 |
|  |  | Average score | 1 | 1 | 1 | 2 | 2 | 1.5 | 1 | 9.5 |
| Molla AA, et al. (52) | 2017 | Temesgen  Gebeyehu (TG) | * | * | * | ** | ** | ** | * | 10 |
|  |  | Ruhama  Gebeyehu (RG) | * | * | * | ** | ** | ** | * | 10 |
|  |  | Average score | 1 | 1 | 1 | 2 | 2 | 2 | 1 | 10 |
| Ebuenyi ID, et al. (53) | 2017 | Temesgen  Gebeyehu (TG) |  |  |  | ** | ** | ** | * | 7 |
|  |  | Ruhama  Gebeyehu (RG) |  |  |  | ** | ** | ** | * | 7 |
|  |  | Average score |  |  |  | 2 | 2 | 2 | 1 | 7 |
| Tadesse WB, et al. (54) | 2019 | Temesgen  Gebeyehu (TG) | * | * | * | ** | ** | ** | * | 10 |
|  |  | Ruhama  Gebeyehu (RG) | * | * | * | ** | ** | ** | * | 10 |
|  |  | Average score | 1 | 1 | 1 | 2 | 2 | 2 | 1 | 10 |

| Madiba S, et al. (55) | 2014 | Temesgen  Gebeyehu (TG) |  |  |  | ** | ** | ** | * | 7 |
| --- | --- | --- | --- | --- | --- | --- | --- | --- | --- | --- |
|  |  | Ruhama  Gebeyehu (RG) |  | * |  | ** | ** | ** | * | 8 |
|  |  | Average score |  | 0.5 |  | 2 | 2 | 2 | 1 | 7.5 |
